# Ratiometric electrochemical detection of kojic acid based on glassy carbon modified MXene nanocomposite†

**DOI:** 10.1039/d3ra05629e

**Published:** 2023-12-19

**Authors:** Gopi Karuppaiah, Aneesh Koyappayil, Anna Go, Min-Ho Lee

**Affiliations:** a School of Integrative Engineering, Chung-Ang University 84 Heuseok-ro, Dongjak-Gu Seoul 06974 Republic of Korea mhlee7@cau.ac.kr

## Abstract

The significance of developing a selective and sensitive sensor for quality control purposes is underscored by the prevalent use of kojic acid (KA) in cosmetics, pharmaceuticals, and food items. KA's utility stems from its ability to inhibit tyrosinase activity. However, the instability of KA and its potential adverse effects have created a pressing need for accurate and sensitive sensors capable of analyzing real samples. This research introduces an electrochemical ratiometric sensor designed to accurately detect KA in actual cosmetic and food samples. The ratiometric sensor offers distinct advantages such as enhanced selectivity, reproducibility, and sensitivity. It achieves this by leveraging the ratio between two output signals, thereby producing reliable and undistorted results. The sensor is constructed by modifying a Glassy Carbon Electrode (GCE) with a nanocomposite consisting of Ti_3_C_2_ MXene, Prussian blue, and gold nanoparticles. The incorporation of MXene and gold nanoparticles heightens sensitivity and reduces impedance. Meanwhile, the Prussian blue signal diminishes proportionally with increasing KA concentration, forming the basis for the ratiometric sensing mechanism. The outcomes of the study reveal a broad linear range (1–600 μM), a low detection limit (1 μM), and strong selectivity for KA. These findings suggest the sensor's potential efficacy in quality control across cosmetics, pharmaceuticals, and food products.

## Abstract 2

Fabrication of MXene nanocomposite-based ratiometric electrochemical sensor for the selective and sensitive determination of Kojic acid (KA).

## Graphical abstract

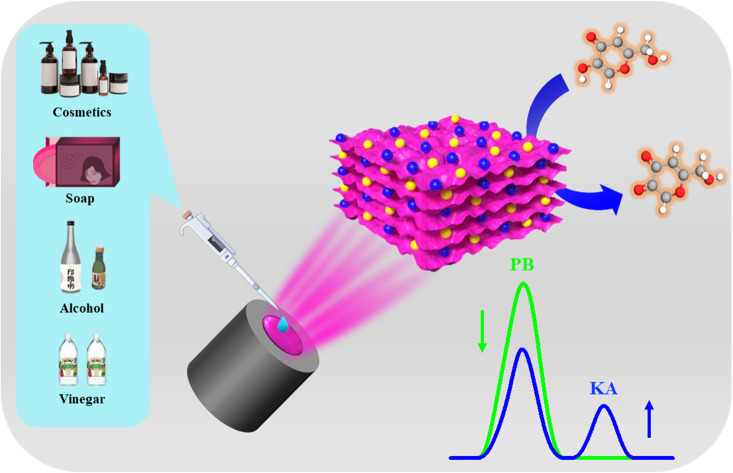

## 1. Introduction

Tyrosinase inhibitors have gained notable attention recently due to their significant role in a variety of cosmetic products and their influence on the food industry, cosmetics, and pharmaceutical sectors.^1,2^ However, concerns persist regarding their safety, potential for cytotoxicity, and stability issues.^3^ Among these inhibitors, kojic acid (KA), a well-known compound, has found widespread application in both the cosmetic and pharmaceutical realms. Its discovery traces back to 1907 when Saito first isolated it from *Aspergillus oryzae* cultures. Yabuta later coined the term “kojic acid” in 1912, while its molecular structure was more comprehensively elucidated in 1924.^4^ KA offers a multitude of advantages, including its abilities in skin lightening, antibacterial action, inhibition of proliferation, anti-inflammatory effects, and antioxidative properties.^5^ In the cosmetic industry, KA is prominently acknowledged for its capacity to inhibit tyrosinase, thereby restricting melanin formation (refer to Fig. S1†). It is a common component in topical treatments for various skin conditions such as melasma, spots, and brown discolouration resulting from post-inflammatory hyperpigmentation (PIH).^5^ Beyond this, KA functions as a safeguard against UV radiation and as a preservative, thereby extending the shelf life of cosmetic products.^6,7^ Combinations of KA with alpha-hydroxy acids are commonly employed in products designed to lighten the skin, combating age spots and freckles. Additionally, the manganese and zinc complexes of KA are attributed to its role as a radioprotective agent against exposure to gamma-rays.^8^ However, the application of KA is accompanied by significant cytotoxicity and instability issues, prompting further investigations to accurately quantify KA concentrations in cosmetic and food samples.^9^ Excessive exposure to KA can lead to adverse effects such as allergic reactions, skin irritation, and genotoxicity.^5^ To adhere to regulatory standards and uphold quality control, it is imperative to precisely monitor the presence and concentration of KA in food, cosmetic, and pharmaceutical products. The Cosmetic Ingredient Review (CIR) has sanctioned a safe usage level of 1% for KA in cosmetic products.^6^

The detection of KA holds significant implications across diverse industries and applications, constituting a crucial field with extensive practical utility. Enhanced KA sensors, boasting improved sensitivity and selectivity, offer the potential to guarantee the safety and quality of products in industries such as food, cosmetics, and pharmaceuticals. Moreover, they offer valuable insights into disease diagnosis and treatment by precisely monitoring KA levels in biological samples like blood, urine, and saliva. This underscores the vital role of KA sensing as a far-reaching research domain. A variety of sensing techniques, including fluorescence-based,^10^ spectrophotometry,^11^ electrochemistry,^12^ high-performance liquid chromatography,^13^ and mass spectrometry,^14^ have been documented for KA determination in food and cosmetic items. Of these, electrochemical methods exhibit high sensitivity, selectivity, cost-effectiveness, and user-friendliness, positioning them as a promising and favoured choice.^15,16^ However, further research is imperative to enhance their potential applications.

**Scheme 1:**
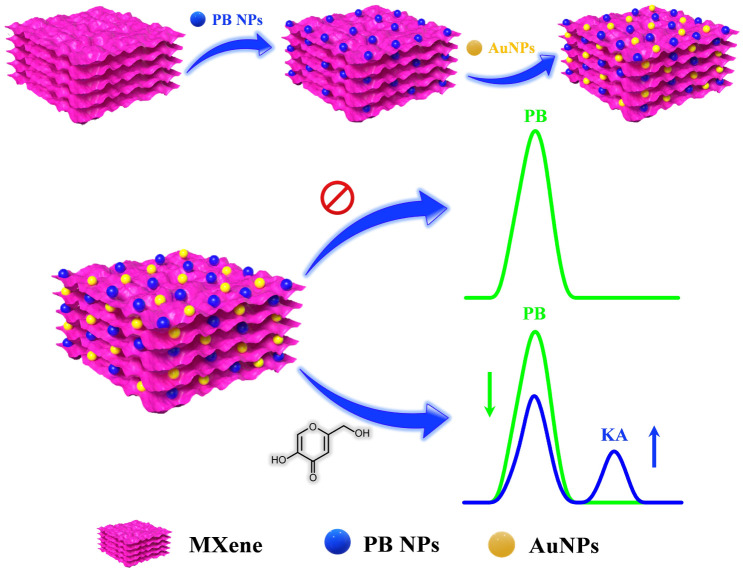
Schematic representation for the fabrication of GCE/MXene/PB/AuNPs modified electrode for electrochemical ratiometric KA determination.

In recent times, notable progress has occurred in the domain of electrochemical sensor applications through the enhancement of 2D materials using metal nanoparticles.^17^ These nanoparticles integrated into 2D materials serve to amplify the electroactive surface and conductivity, thereby augmenting the sensitivity of electrochemical detection. A specific category of 2D materials, known as MXenes, composed of transition metal carbides/nitrides has attracted considerable scientific attention due to their distinctive chemical and electronic properties, viable synthesis methods, and the capacity for controlled property modulation by adjusting the proportions of M and/or X elements.^18^ The concept of electrochemical ratiometric sensors, introduced in 2013,^19^ signifies a significant advancement in the realm of electrochemical biosensors and is presently gaining prominence. Traditional electrochemical sensors typically rely on the absolute magnitude of a solitary signal for both identification and quantification purposes. Conversely, ratiometric electrochemical sensors measure the ratio of electrochemical signals, enabling the determination and quantification of the targeted analyte. This approach yields heightened selectivity, reproducibility, accuracy, and sensitivity, which are essential prerequisites for modern electrochemical sensors.^20–23^ In this study, we present an on-off mode ratiometric electrochemical sensor tailored for the precise and sensitive detection of KA (Scheme 1). This sensor utilizes a nanocomposite consisting of MXene, Prussian blue, and gold nanoparticles to modify a glassy carbon electrode (GCE). Notably, there exists no prior record of a ratiometric sensor being employed for KA detection in the existing literature.

## 2. Materials and methods

### 2.1. Chemicals and reagents

Potassium bromide, iron(iii) ferrocyanide (Prussian blue), potassium hexacyanoferrate(iii) (ACS reagent, ≥99%), kojic acid (≥98.5%, HPLC), magnesium chloride (anhydrous, ≥98%), glucose, sodium chloride (ACS reagent, ≥99.0%), sodium bicarbonate (ACS reagent, ≥99.7%), trisodium citrate dihydrate, sulfuric acid (ACS reagent, 95.0–98.0%), hydrochloric acid (ACS reagent, 37%), and Nafion perfluorinated resin solution were purchased from Sigma-Aldrich (Korea). Potassium chloride (>99.5%) was purchased from Kanto Chemicals Co., Inc. Gold colloidal nanoparticles with a diameter of 40 nm were sourced from Bore Da Biotech. Phosphate-buffered saline at pH 7.4 was acquired from Thermo Fisher Scientific. Calcium hydroxide was purchased from Daejung Chemicals in Korea, while anhydrous ethyl alcohol with a purity of 99.9% was obtained from Samchun Chemical, Korea. The working glassy carbon electrode (GCE), a spiral platinum counter electrode, and Ag/AgCl reference electrodes were procured from BASi. All the chemicals and buffers were prepared with deionized water with a resistance not less than 18.2 MΩ cm.

### 2.2. Synthesis of Ti_3_C_2_T_*x*_ MXene from the MAX phase

**Fig. 1:**
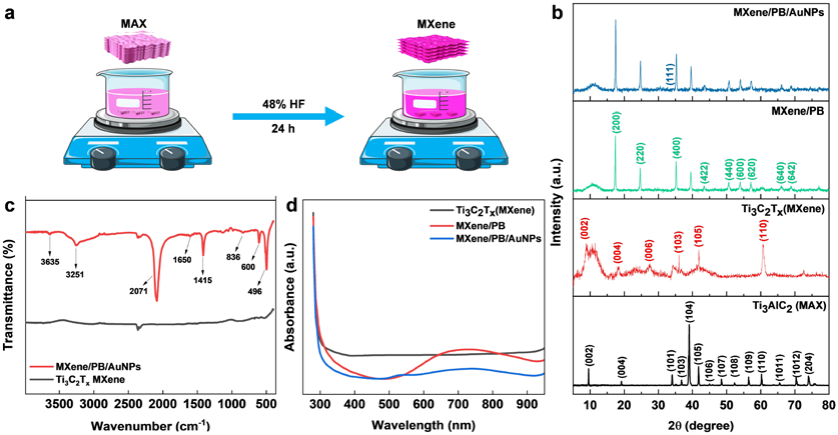
(a) Schematic diagram of the synthesis of Ti_3_C_2_T_*x*_ MXene from Ti_3_AlC_2_ MAX phase by acid etching method. (b) XRD pattern of Ti_3_AlC_2_ MAX phase, Ti_3_C_2_T_*x*_ MXene, MXene/PB, and MXene/PB/AuNPs nanocomposite. (c) FT-IR spectra of MXene and MXene/PB/AuNPs, and (d) UV-vis spectrum of MXene, MXene/PB, and MXene/PB/AuNPs.

The Ti_3_C_2_T_*x*_ MXene was prepared by the conventional etching of Al atoms from the Ti_3_AlC_2_ MAX phase using HF acid (Fig. 1a).^24^ Briefly, 48% HF was slowly added to 5.0 g of the Ti_3_AlC_2_ MAX phase followed by magnetic stirring for 24 hours at room temperature. The resulting MXene suspension was collected and then washed repeatedly with DI water until the suspension reached near neutral pH (pH ≥ 6). The suspension was then sonicated, centrifuged, washed with absolute ethanol, and dried at room temperature.

### 2.3. Fabrication of GCE/MXene/PB/AuNPs/NF electrode

To examine the electrochemical performance of the electrochemical sensor, the working electrode was fabricated. Initially, a glassy carbon electrode (GCE) underwent polishing using 0.05 μm alumina slurry, followed by sonication in deionized water and ethanol. Subsequently, it was dried using a nitrogen stream. The working electrode was formed *via* the drop-casting technique. Briefly, 5 mg of the synthesized MXene and 2 mg of Prussian blue were dispersed in 1 mL of distilled water through continuous vortexing and sonication for 15 minutes, resulting in PB/MXene solution. Then, 100 μL each of gold nanoparticles and PB/MXene were sonicated together for 20 minutes to create the MXene/PB/AuNPs composite. Ultimately, 4 μL of the assembled GCE/MXene/PB/AuNPs nanocomposite was drop-cast onto the GCE surface and left to dry at room temperature for an hour. A final surface coating using 1% Nafion was applied to the electrode to prevent nanoparticles leaching into the electrolyte during analysis. For the control experiment, GCE, GCE/MXene, GCE/MXene/PB, GCE/MXene/PB/AuNPs, GCE/PB/AuNPs, and GCE/MXene/PB/AuNPs/NF were prepared following the aforementioned procedure. Prior to electrochemical measurements, all prepared electrodes underwent stabilization through repeated cycling in pH 7.4 (0.1 M) until a consistent voltammogram was achieved. Subsequently, the GCE/MXene/PB/AuNPs/NF electrode underwent optimization for optimal performance (see ESI†).

### 2.4. Instrumentation

Field emission transmission electron microscopy (FE-TEM) measurements were made on a JEOL JEM-F200 instrument. Fourier-transform infrared (FTIR) spectra were obtained using a Thermo Scientific Nicolet 6700 with sample pellets prepared by using ultrapure KBr. X-ray diffraction (XRD) analysis was performed on Bruker-AXS New D8-Advance X-ray diffractometer with Cu Kα radiation. Ultraviolet-visible (UV-vis) spectroscopy analysis was performed on a Biotek Synergy H1 microplate reader using 96-well clear polystyrene microplates. X-ray photoelectron spectroscopy analysis was performed on Thermo Fisher Scientific K-alpha^+^ X-ray photoelectron spectroscope.

Electrochemical analyses were executed using a CHI 660E electrochemical workstation manufactured by CH Instruments, Inc. The analyses included cyclic voltammetry (CV), Differential Pulse Voltammetry (DPV), and impedance analysis. These experiments were conducted using a standard three-electrode setup: a Glassy Carbon Electrode (GCE) modified for the working electrode, a platinum counter electrode in spiral form, and an Ag/AgCl reference electrode. The initial stage of electrochemical assessment involved the gradual modification of the nanocomposite on the GCE surface. This was achieved by employing a CV within a pH 7.4 PBS solution. The potential window ranged from −0.5 to 1.0 V, and the scan rate was fixed at 0.05 V s^−1^. DPV was employed to investigate the dose responses of different concentrations of KA. The DPV parameters were configured as follows: a scan rate of 20 mV s^−1^, a pulse amplitude of 50 mV, a pulse width of 0.06 s, and a pulse period of 0.5 s. Impedance analysis spanned a frequency spectrum of 1 to 100 000 Hz. Furthermore, the sensitivity and selectivity of the modified electrode were assessed *via* DPV conducted in a pH 7.4 PBS.

## 3. Results and discussion

### 3.1. Material characterization

Preliminary spectral analysis on the synthesized Ti_3_C_2_T_*x*_ MXene and MXene/PB/AuNPs nanocomposite was performed by XRD, FT-IR, and UV-vis spectroscopy. The crystal structure of the Ti_3_AlC_2_ MAX phase, as-synthesized Ti_3_C_2_T_*x*_ MXene and the MXene/PB/AuNPs nanocomposite was analysed by X-ray diffraction analysis (Fig. 1b). The XRD pattern of the Ti_3_C_2_T_*x*_ MAX phase was consistent with the pattern of bulk Ti_3_AlC_2_ (PDF #52-0875). The HF treatment resulted in a decrease in the structural order as well as crystallinity of the Ti_3_AlC_2_ MAX phase. The most intense peak at 39.2°, corresponding to the (1 0 4) crystallographic plane was absent in the XRD pattern of Ti_3_C_2_T_*x*_ MXene, confirming the removal of the Al layer of atoms from the MAX phase.^25^ Besides, the XRD patterns of the synthesized MXene were consistent with the simulated spectrum of Ti_3_C_2_OH.^26^ In the XRD spectrum of MXene/PB, the peaks corresponding to 2*θ* = 17.4°, 24.7°, 35.2°, 39.5°, 43.5°, 50.6°, 53.9°, 57.1°, 66.1°, and 68.8° were assigned to the lattice planes (2 0 0), (2 2 0), (4 0 0), (4 2 0), (4 2 2), (4 4 0), (6 0 0), (6 2 0). (6 4 0), and (6 4 2) reported for Fe_4_[Fe(CN)_6_]_3_ (Prussian blue) cubic crystal system, which is in agreement with the standard XRD pattern (PDF #00-052-1907).^27^

This result also confirmed the Prussian blue particles inside the nanocomposite. When gold nanoparticles were introduced into the nanocomposite, an additional low-intensity peak was observed at 38.1°, which was assigned to the (1 1 1) plane, matching the JCPDS patterns of pure crystalline gold (JCPDS no. 04-0784). Besides, the (1 1 1) plane was assumed to be the most predominant. The average crystalline size of the Ti_3_C_2_T_*x*_, Prussian blue, and gold nanoparticles was calculated by using the Debye–Scherrer equation (eqn (1)).

1 *D* = *Kλ*/*β* cos *θ*

where *D* is the crystalline size in nm, *λ* is the X-ray wavelength (0.15406 nm), *K* is the Scherrer constant (0.9), *β* is the full width at half maximum (FWHM) in radians, and *θ* is the peak position in radians. The crystalline size of the Ti_3_C_2_T_*x*_ MXene, PB NPs, and AuNPs was calculated to be. 4.72 nm, 28.67 nm, and 2.64 nm respectively. FTIR analysis was performed to gain further insights into the chemical functionalities of the nanocomposite. As shown in Fig. 1c, from the FTIR spectra of MXene and MXene/PB/AuNPs nanocomposite, the absorption band for MXene around 3545 cm^−1^ was likely due to the absorbed water and indicates the hydrophilic nature of the MXene.^28^ The peaks around 603 and 1529 cm^−1^ were assigned to Ti–O and C–F respectively. The band at 2091 cm^−1^ was assigned to the characteristic CN stretching of Fe–CN in Prussian blue.^29^ The UV-vis spectrum (Fig. 1d) of Ti_3_C_2_T_*x*_ MXene exhibited no characteristic absorption above 300 nm, consistent with the delaminated thin films of Ti_3_C_2_T_*x*_,^30^ whereas the MXene/PB has shown an intense band centered around 720 nm originating from the charge transfer between Fe^2+^ and Fe^3+^ inside Prussian blue, and the MXene/PB/AuNPs have shown an additional peak around 530 nm, characteristic of the surface plasmon resonance (SPR) peak of AuNPs.

**Fig. 2:**
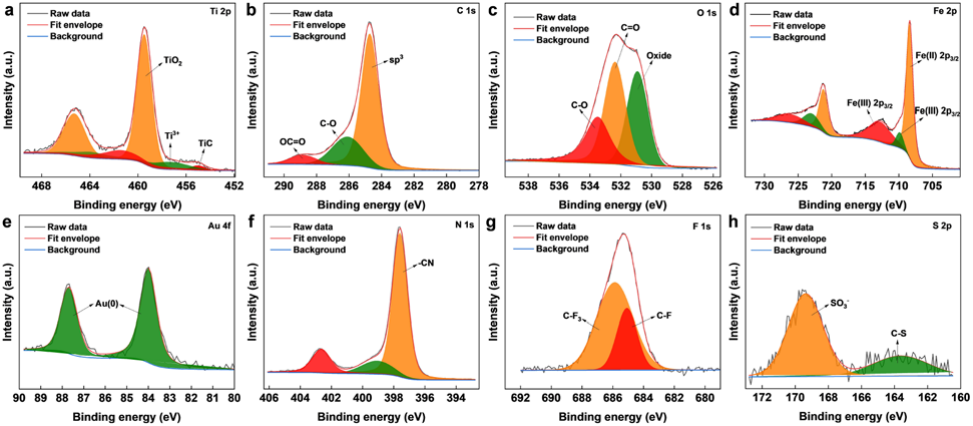
XPS deconvoluted core-level peaks for (a) Ti 2p, (b) C 1s, (c) O 1s, (d) Fe 2p, (e) Au 4f, (f) N 1s, (g) F 1s, and (h) S 2p.

The surface chemical composition of the as-synthesized Ti_3_C_2_T_*x*_ MXene and MXene nanocomposite was analysed by X-ray photoelectron spectroscopy (XPS) (Fig. 2 and S2†). From the survey spectrum of the nanocomposite (Fig. S3†), the presence of elements titanium, carbon, oxygen, nitrogen, gold, iron, fluorine, and sulphur was confirmed, which was also supported by the elemental mapping results. The deconvoluted spectrum of Ti 2p (Fig. 2a) was composed of peaks cantered around ∼454.6, and ∼457.6 eV corresponding to the Ti–C bond and Ti^3+^ respectively.^31^ The peak centered around ∼459 eV corresponds to Ti^4+^, probably due to TiO_4_, which also explains the oxide peak in the deconvoluted spectrum of O 1s. The signals from C 1s (Fig. 2b) cantered at ∼284.7, ∼286.2, and ∼288.6 eV were attributed to sp^3^ carbon, C–O groups, and OC=O. The core-level spectrum of O 1s (Fig. 2c) comprised peaks around ∼533.5, ∼532.3, and ∼530.8 eV corresponding to CO, C=O, and oxide. As shown in Fig. 2d, the core-level spectrum of Fe 2p was deconvoluted into peaks corresponding to the binding energies of Fe(ii)2p_1/2_, Fe(iii)2p_1/2_, Fe(ii)2p_3/2_, and Fe(iii)2p_3/2_, also confirmed the co-existence of Fe^2+^ and Fe^3+^.^32^ The XPS spectrum of Au 4f (Fig. 2e) fitted by two components around ∼84, and ∼87 eV, attributed to the Au (0) from the AuNPs.^33^ The deconvoluted core-level spectra of N 1s (Fig. 2f) consist of three peaks at 402.6, 399.2, and 397.5 eV, identifying the existence of the nitrile group of [Fe(CN)_6_]^4−^.^34^ The fluorine fraction of the nanocomposite (Fig. 2g), which was contributed mainly by Nafion was deconvoluted into peaks corresponding to C–F and CF_3_,^35^ whereas the sulphur fraction originating from Nafion was deconvoluted into peaks (Fig. 2h) corresponding to C–S, and SO_3_^−^.^36^

### 3.2. Morphology

**Fig. 3:**
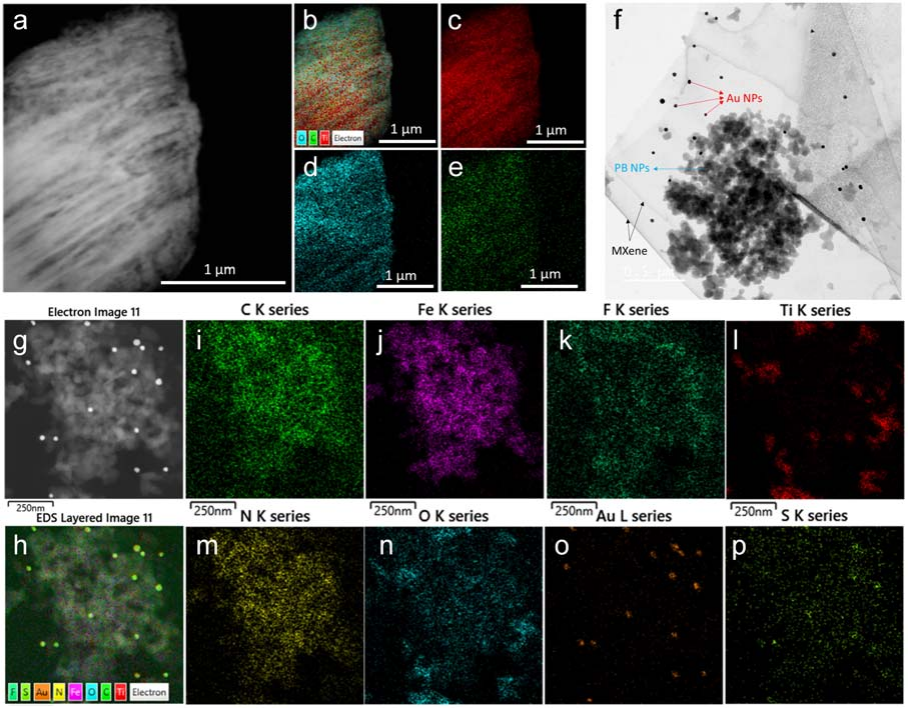
(a) FE-TEM image of Ti_3_C_2_T_*x*_ MXene, (b) EDS layered image of Ti_3_C_2_T_*x*_, and EDS elemental mapping of (c) titanium, (d) oxygen, and (e) carbon. (f) FE-TEM image of Ti_3_C_2_T_*x*_/PB/AuNPs nanocomposite showing the distribution of PB NPs, and AuNPs over MXene nanolayers. (g) FE-TEM image of Ti_3_C_2_T_*x*_/PB/AuNPs/NF nanocomposite, (h) EDS layered image of Ti_3_C_2_T_*x*_/PB/AuNPs/NF, and EDS elemental mapping of (i) titanium, (j) carbon, (k) oxygen, (l) iron, (m) nitrogen, (n) gold, (o) sulphur, and (p) fluorine.

The morphology of the as-synthesized Ti_3_C_2_T_*x*_ MXene and MXene nanocomposite was analysed by FE-TEM analysis. As shown in Fig. 3a, a signature accordion-like structure was observed for the Ti_3_C_2_T_*x*_ MXene. The composition of the MXene and the nanocomposite was also confirmed by energy-dispersive X-ray spectroscopy (EDS) analysis. EDS elemental mapping of MXene confirmed the homogenous distribution of elements titanium, carbon, and oxygen on MXene layers (Fig. 3b–e). The FE-TEM analysis also confirmed the distribution of Prussian blue and gold nanoparticles on the MXene nanolayers (Fig. 3f–h). From the HR-TEM image (Fig. S4†), the diffraction fringe with an interlayer spacing of 0.991 nm is indexed to the (0 0 2) lattice plane of Ti_3_C_2_T_*x*_ MXene.^37^ As shown in Fig. 3i–p and S5,† the elemental analysis on the nanocomposite confirmed the distribution of elements titanium, carbon, oxygen, nitrogen, gold, iron, fluorine, and sulphur, which was consistent with the XPS analysis.

### 3.3. Preparation and optimization of electrocatalytic properties of GCE/MXene/PB/AuNPs/NF

The electrochemical performance of the MXene nanocomposite for KA detection was investigated by fabricating the MXene nanocomposite modified GCE (GCE/MXene/PB/AuNPs/NF). The prepared GCE/MXene/PB/AuNPs/NF electrode was stored at 4 °C in the refrigerator before use. AuNPs and MXene were well reported to increase the electroactive surface area, thereby increasing the sensitivity of electrochemical sensors. In this work, MXene also served to entrap and stabilize the nanoparticles. The Nafion coating increased the sensitivity by preventing the leaching out of the nanomaterials into the electrolyte solution. The electrode was initially optimized for the optimal performance of the sensor (Fig. S6, ESI†).

### 3.4. Sensing mechanism

**Fig. 4:**
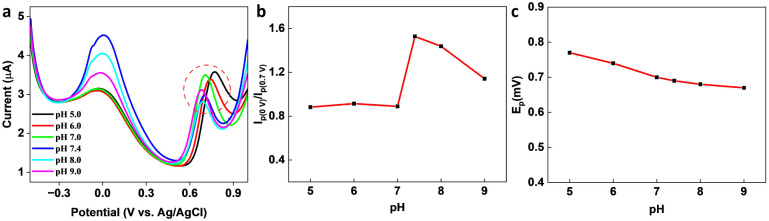
(a) pH dependency studies on the GCE/MXene/PB/AuNPs/NF. (b) Plot of the ratio of peak currents at 0 V and 0.7 V (*I*_p (0 V)_/*I*_p (0.7 V)_) *vs.* pH, and (c) plot of peak potential *vs.* pH.

In the presence of KA, the oxidation peak around 0 V decreased and another oxidation peak appeared around 0.7 V (*vs.* Ag/AgCl) in the DPV curve, thereby forming the basis (*I*_p (0 V)/_*I*_p (0.7 V)_) of the on-off mode ratiometric sensor. The impact of pH on the ratiometric response of KA detection using GCE/MXene/PB/AuNPs was investigated across a pH range of 5–9 (Fig. 4a). The oxidation peak current at 0 V, responsible for the Prussian blue oxidation, decreased with a decrease in pH, whereas the oxidation peak at 0.7 V, which is due to KA oxidation, varied with pH changes and a maximum was observed at pH 5. However, since *I*_p (0 V)_/*I*_p (0.7 V)_ ratio was found to be higher at pH 7.4 (Fig. 4b), pH 7.4 was selected for the fabrication of the ratiometric sensor. As shown in Fig. 4c, the peak potential (*E*_p_) for KA oxidation at 0.7 V shifted towards the negative as pH increased, indicating the involvement of protons in the current-limiting electrode process. Therefore, a mechanism for the ratiometric sensor was proposed (Fig. S7†), which involves two key electrochemical processes occurring at the electrode surface. The first process involves the reduction of Prussian blue to Prussian white, which is responsible for the decrease in oxidation current around ∼0 V, while the second process entails the oxidation of KA and is responsible for the increase in oxidation current around ∼0.7 V. These chemical transformations provide the foundation for the ratiometric sensor, allowing precise measurement of KA in a reliable and consistent manner using the GCE/MXene/PB/AuNPs/NF electrode.

### 3.5. Performance of the electrochemical ratiometric KA sensor

**Fig. 5:**
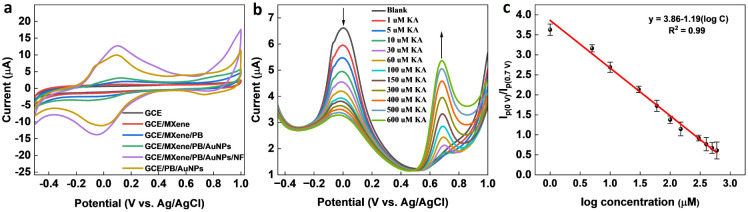
(a) CV responses of bare GCE, GCE/MXene, GCE/MXene/PB, GCE/MXene/PB/AuNPs, GCE/PB/AuNPs, and GCE/MXene/PB/AuNPs/NF towards 100 μM KA. (b) DPV responses of GCE/MXene/PB/AuNPs/NF towards increasing concentrations of KA, and (c) the corresponding linear calibration plot of *vs.* logarithmic concentration of KA.

To optimize the performance of the ratiometric sensor platform, DPV responses of GCE, GCE/MXene, GCE/MXene/PB, GCE/MXene/PB/AuNPs, GCE/PB/AuNPs, and GCE/MXene/PB/AuNPs/NF was recorded in the presence of 100 μM KA. As shown in Fig. 5a, the highest peak current response was shown by the GCE/MXene/PB/AuNPs/NF electrode modification, and consequently selected for the sensor fabrication. The ratiometric KA sensor was fabricated by injecting increasing concentrations of KA into 20 mL of PBS (pH 7.4) and the DPV response was recorded by using the GCE/MXene/PB/AuNPs/NF electrode. A gradual decrease in the peak current around 0 V, and an increase in the peak current around 0.7 V was observed with an increase in the concentration of KA (Fig. 5b). The ratio of the peak current responses (*I*_p (0 V)/_*I*_p (0.7 V)_) was then plotted against the logarithmic concentration of KA. A good linear relationship was observed (*R*^2^ = 0.99) between the peak current ratio and the logarithmic KA concentration over the range of 1–600 μM with a limit of detection of 1 μM (Fig. 5c). The results also demonstrated the superior performance of our reported sensor in comparison with the previously reported electrochemical KA detection methods (Table 1).

**Table 1:** Literature comparison of various electrochemical sensors reported for KA detection

Electrode modification	Sensing technique	Linear range (μM)	LOD (μM)	Real sample	Ref.
CuO/Fe_2_O_3_-Chi/GCE	Amperometry	0.2–674	0.08	Cosmetics, sauce, vinegar	38
MIP/GCE	DPV	0.01–0.2	0.003	Cosmetics samples	39
PVP/ABPE	LSV	1.0–100	0.5	Sauce, vinegar	40
Pre-anodized SPCE	SWV	Up to 260	0.17	Cosmetics samples	41
EPPG electrode	LSV	0.75–15	0.23	—	42
MWNT/ARS/GCE	Amperometry	0.4–6	0.1	Sauce, vinegar	43
RGSs/GCE	LSV	0.01–0.14	—	—	44
GCE	SWV	35–250	7.84	Cosmetics samples	45
MWCNTs-SPCE	DPV	20–5000	16	Vinegar	12
PGA/GCE	CV	8–660	0.8	Bean paste, rice wine	46
Gr-Pt/CS/GCE	DPV	0.2–1000	0.2	Sauce, vinegar	47
V_2_O_5_/NPs/HMIHPF_6_/CPE	SWV	0.08–500	0.02	Bean paste, rice wine, vinegar	48
CPE/MgO-NPs-/M_3_BIBr	DPV	0.1–700	0.04	Edible oil, chilli sauce	49
TiO_2_/Fe_3_O_4_/MWCNTs/IL-CPE	DPV	0.5–300	0.2	Edible oil, chilli sauce	50
NiO/NPs/CPE	SWV	5–600	0.8	Urine, serum, vinegar	51
(Hpy)(PF_6_)/N-CQDs/Fe_3_O_4_/CPE	DPV	0.03–300	0.008	Tomato sauce, vinegar	52
[Fe(HL)_2_Cl_2_]nanocomplex-IL/CPE	DPV	0.3–237	0.09	Edible oil, chilli sauce	53
MXene/PB/AuNPs/NF	DPV	1–600	1	Cosmetic samples, vinegar	This work

### 3.6. Selectivity and reproducibility of the KA sensor

**Fig. 6:**
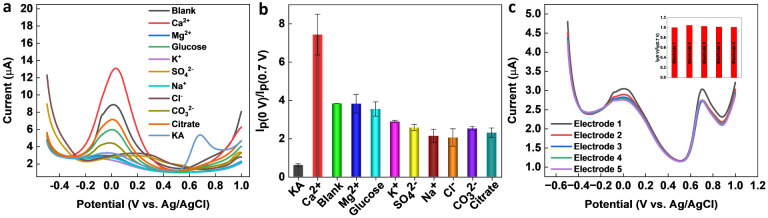
(a) Interference study on the GCE/MXene/PB/AuNPs/NF electrode with 600 μM KA and 1 mM of interfering ions and molecules (Ca^2+^, Mg^2+^, K^+^, SO_4_^2−^, Na^+^, Cl^−^, CO_3_^2−^, citrate, and glucose). (b) The corresponding plot of peak current ratio *vs.* interferents. (c) Reproducibility test using five GCE/MXene/PB/AuNPs/NF electrodes (inset shows the corresponding plot of peak current ratio *vs.* electrodes).

Selectivity is a crucial parameter for the successful implementation of sensors. A selective sensor can distinguish between the target analyte and other interfering compounds present in the sample matrix, thereby ensuring accurate quantification. The importance of the selectivity for a KA sensor cannot be overemphasized, since inaccurate quantification of KA can result in false positives or false negatives, leading to significant consequences in food safety, cosmetic quality control, and disease diagnosis and treatment. A selective KA sensor can provide accurate results, ensuring the safety and quality of food, cosmetic, and pharmaceutical products, as well as contributing to the development of disease diagnosis and treatment methods. Various interfering compounds and ions such as ethanol, glucose, citrate, Na^+^, K^+^, Mg^2+^, Cl^−^, SO_4_^2−^, CO_3_^2−^, and Ca^2+^ can co-exist in food, pharmaceutical, cosmetic, and biological samples. Therefore, the selectivity of the ratiometric KA sensor was performed in the presence of a higher concentration (1 mM) of interfering molecules and ions. From Fig. 6a and b, it is evident that the tested molecules and ions produced negligible interference on the final response of the ratiometric sensor. The reproducibility of the sensor was evaluated by fabricating five independent electrodes and then testing a known KA concentration within the observed linear range of the sensor. As shown in Fig. 6c, the sensor responses were reproducible with an RSD of 1.8%.

### 3.7. Real sample analysis

To validate the practical application of the developed sensor, real samples, including cosmetics (Kojic acid soap) and food products (Apple cider vinegar) were tested for the presence of KA. A simple and efficient sample preparation method was used to prepare the samples (ESI†), and the sensor response toward KA was then measured using the optimized conditions established in the previous sections. The results indicated the presence of KA in the analysed cosmetic sample; however, no KA was detected in the food product. To further assess the accuracy and feasibility of the KA sensing platform, the determination of KA was conducted in real samples by a standard addition method. Three different concentrations of the KA within the linear calibration range of the sensor were prepared in real samples, tested with the sensor, and the percentage recoveries were calculated. As shown in Table 2, the observed recoveries were in the range of 96.2–104.1, and the relative standard deviation (RSD) was in the range 3.1–4.6%. The results indicated reliable detection of KA from the tested cosmetic and food samples. The developed sensor exhibited excellent selectivity towards KA, as there were no significant interferences from other potential interfering compounds present in the samples, confirming the accuracy and reliability of the developed sensor for the determination of KA in real samples. Overall, the developed sensor shows great potential for use as a rapid and efficient tool for monitoring KA levels in cosmetic and food products, thereby ensuring product quality and safety.

**Table 2:** Recovery experiment on the GCE/MXene/PB/AuNPs/NF electrode using real samples

Sample	Detected (μM)	Spiked (μM)	Found (μM)	Recovery (%)	RSD (*n* = 3, %)
Kojic acid soap	19.5	50	69.9	100.5	3.7
		100	121.7	101.8	3.6
		200	225.6	102.7	4.1
Apple cider vinegar	—	50	48.1	96.2	4.6
		100	102.5	102.5	3.1
		200	208.3	104.1	4.3

## 4. Conclusions

In conclusion, this study presents a new ratiometric electrochemical sensor with outstanding potential for detecting and monitoring KA in real samples. It involves a GCE modified with a nanocomposite of MXene, Prussian blue, and gold nanoparticles. The GCE/MXene/PB/AuNPs/NF sensor exhibits excellent sensitivity, reproducibility, and ability to detect KA in the presence of other interfering ions and molecules, making it a promising sensor for a wide range of analytical applications. Moreover, the sensor's performance in detecting KA in commercial food, and skin-lightening products demonstrates its potential for food, beverage, and cosmetic sample monitoring. The wide linear response (1–600 μM), low detection limit (1 μM), high selectivity, and sensitivity highlight the sensor's broad implications and promising capabilities for various industries and scientific fields, including biomedical, environmental, and chemical analysis. Furthermore, the electrochemical responses, derived from the modification of an atomically smooth glassy carbon electrode (GCE) surface, present an exciting avenue for extending these findings to the realm of practical applications on portable P–O–C devices.

## Author contributions

Gopi Karuppaiah: methodology, investigation, data curation, formal analysis. Aneesh Koyappayil: conceptualization, methodology, visualization, writing the original draft & editing. Anna Go: methodology. Min-Ho Lee: funding acquisition, project administration, supervision, writing – review & editing.

## Conflicts of interest

There are no conflicts to declare.

## Supplementary Material

RA-013-D3RA05629E-s001
