# Supplementary material for: Ratiometric electrochemical detection of kojic acid based on glassy carbon modified MXene nanocomposite
Source: RSC Adv. 2023 Dec 19;13(50):35766–72. doi: 10.1039/d3ra05629e (PMC10728780; doi:10.1039/d3ra05629e)
Supplement: RA-013-D3RA05629E-s001 [file RA-013-D3RA05629E-s001.pdf]

Electronic Supplementary Material (ESI) for RSC Advances.
This journal is © The Royal Society of Chemistry 2023

Supporting information

Ratiometric electrochemical detection of Kojic acid based on Glassy carbon modified MXene nanocomposite

Gopi Karuppaiah [a ‡] , Aneesh Koyappayil [a ‡], Anna Go [a], Min-Ho Lee [a *]

[a] School of Integrative Engineering, Chung-Ang University, 84 Heuseok-ro, Dongjak-Gu, Seoul 06974, Republic of Korea

‡ These authors contributed equally to this work.

*Corresponding author:

mhlee7@cau.ac.kr; Tel: +82-2-820-5503; Fax: +82-2-814-2651

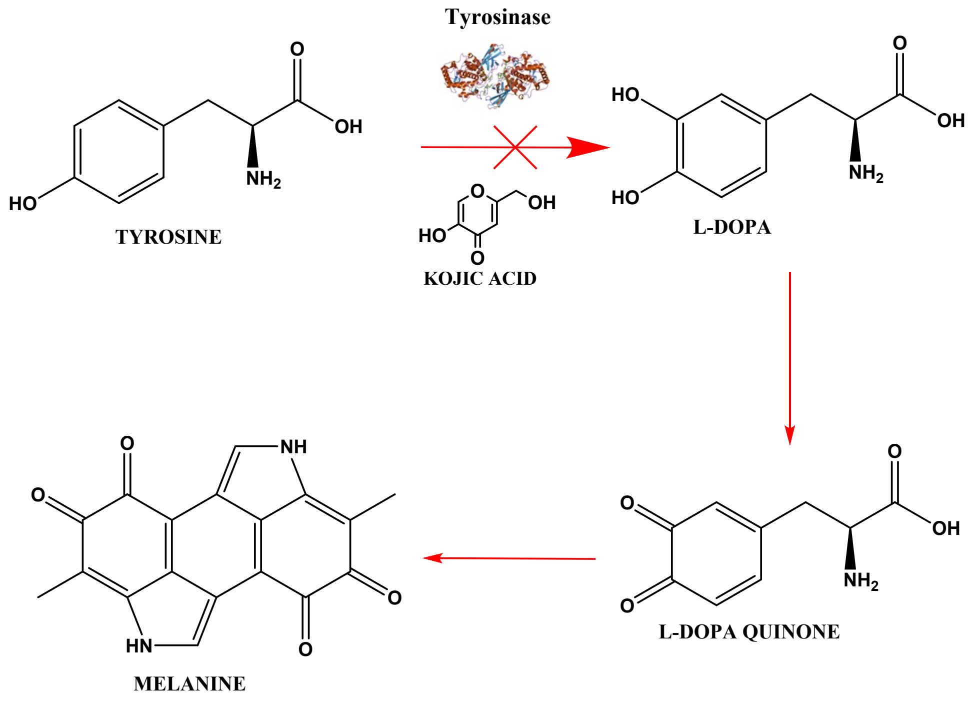

Fig. S1 Schematics of the Tyrosinase inhibition of Kojic acid leading to the decreased production of Melanin.

XPS characterization of $Ti_3C_2T_x$ MXene

X-ray photoelectron spectroscopy (XPS) was employed to analyze the chemical composition of the as-synthesized $Ti_3C_2T_x$ MXene. The survey spectrum of the MXene, as illustrated in Fig. S2a, confirmed the presence of Titanium, Carbon, and Oxygen, which was further supported by the EDS elemental mapping (Figure. S1). Deconvolution of the core-level spectrum of Ti 2p (Fig. S2b) revealed peaks at approximately ~454.6, ~457.6 eV, and ~459 eV corresponding to Ti-C bond, Ti^{3+}, and Ti^{4+}, respectively[1]. The deconvoluted spectrum of C 1s (Fig. S2c) comprised peaks centered around ~281.6, 282.4, ~284.7, ~286.2, and ~288.6 eV, assigned to C-Ti, C-Ti-O, sp3 carbon, C-O groups, and OC=O[1]. The deconvoluted core-level spectrum of O 1s (Fig. S2d) showed peaks centered around ~533.5, ~532.3, and ~530.8 eV, which corresponded to C-O, C=O, and Oxide.[1,2]

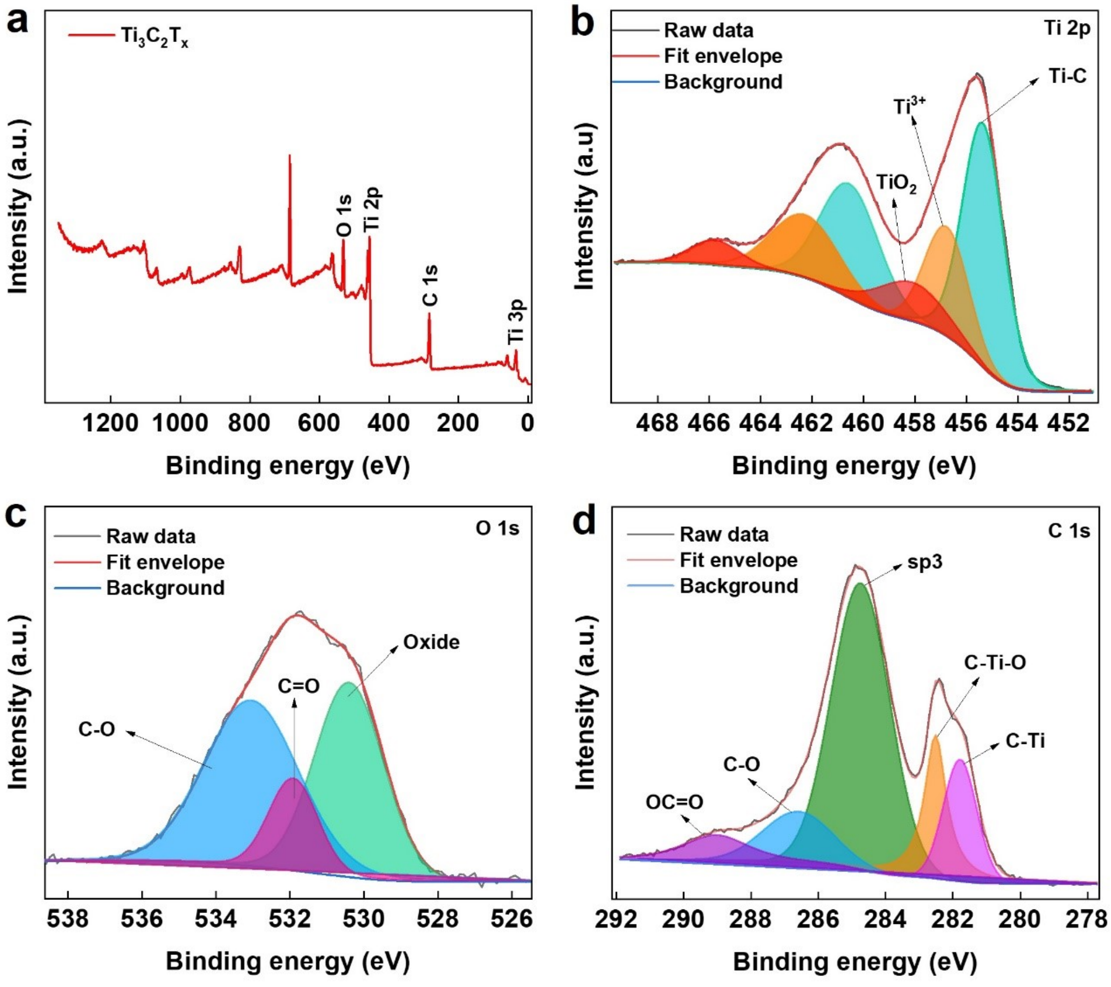

Fig. S2 (a) XPS survey spectrum of the as-synthesized Ti_3C_2 MXene, deconvoluted core-level peaks for (b) Titanium 2p, (c) Oxygen 1s, and (d) Carbon 1s.

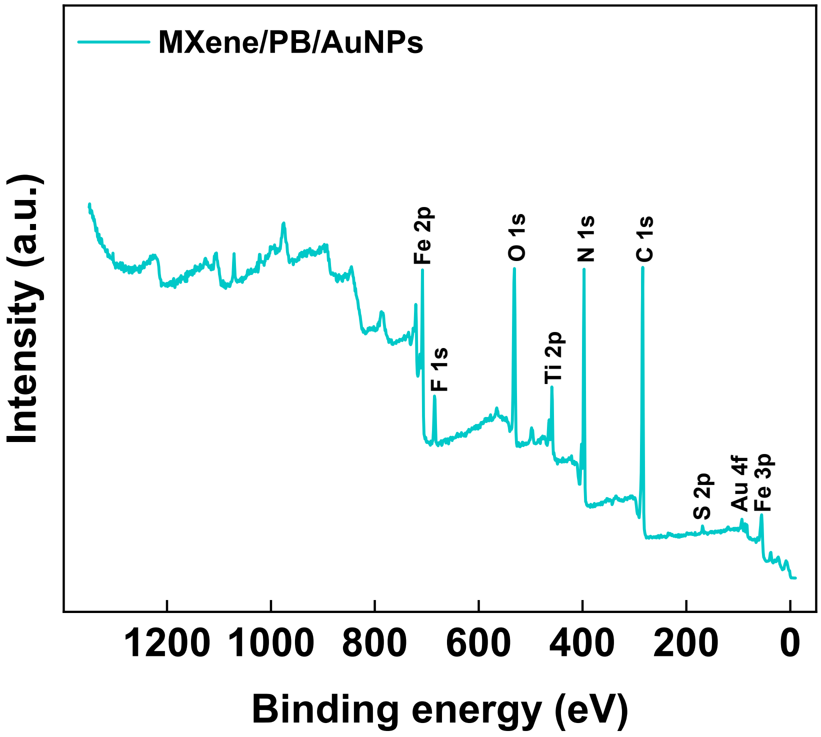

Fig. S3 XPS Survey spectrum of $Ti_3C_2T_x$ MXene/PB/AuNPs nanocomposite.

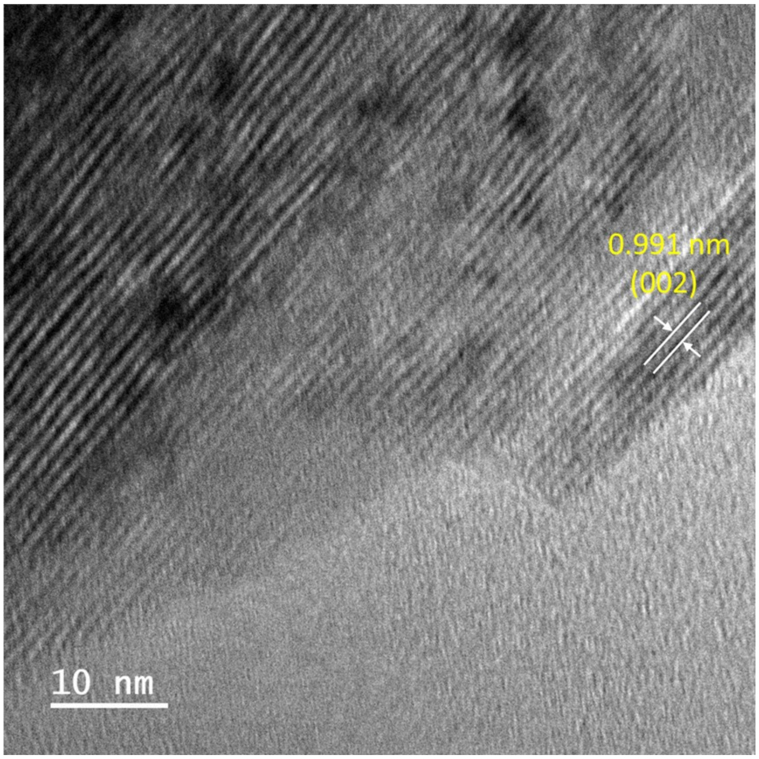

Fig. S4 HR-TEM image of $Ti_3C_2T_x$ MXene showing the interlayer spacing corresponding to the (0 0 2) lattice plane.

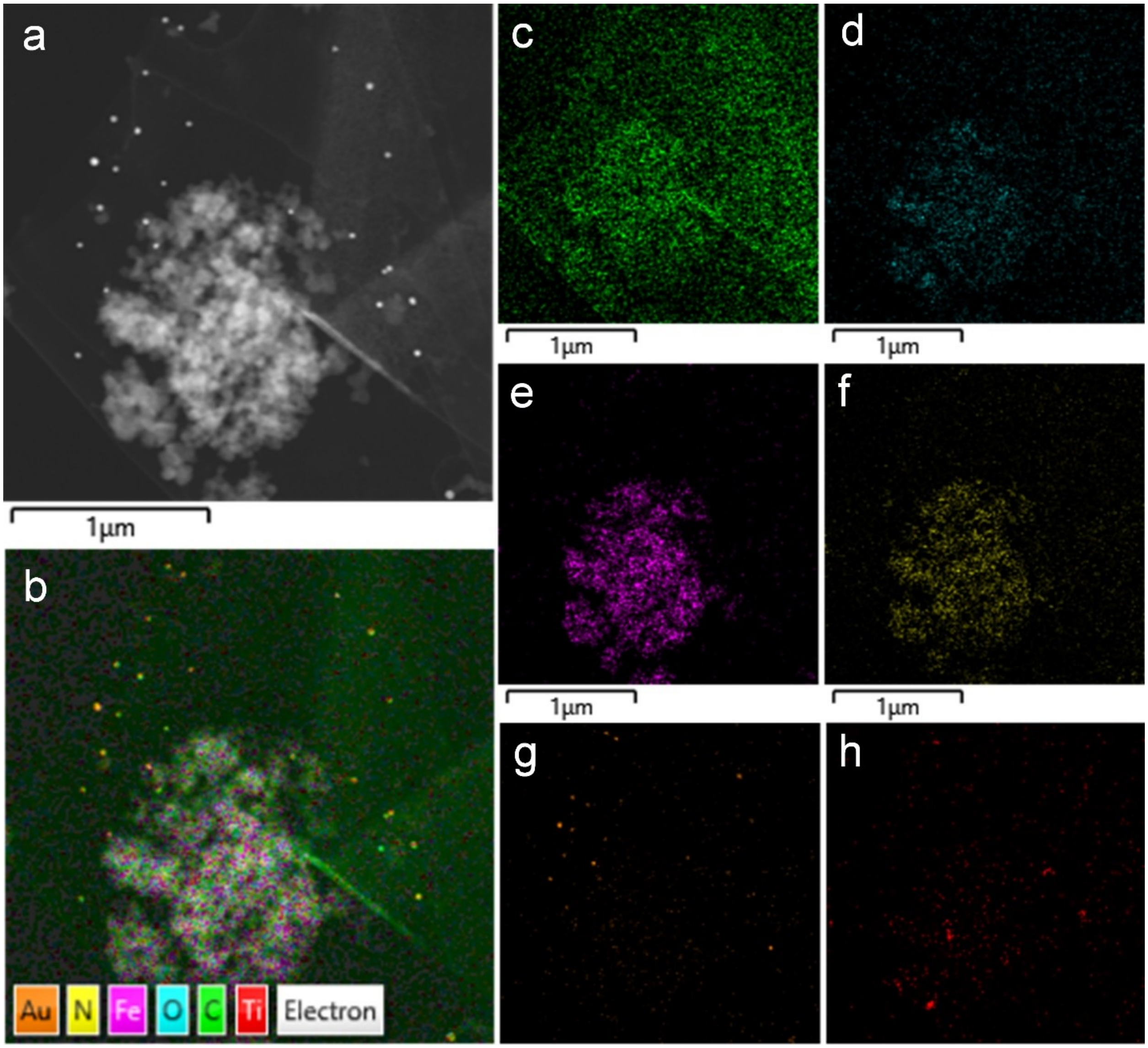

Fig. S5. (a) FE-TEM image of $Ti_3C_2T_x$ MXene/PB/AuNPs, (b) EDS layered image, Elemental mapping of (c) Carbon, (d) Oxygen, (e) Iron, (f) Nitrogen, (g) Gold, and (h) Titanium.

Optimization of the electrochemical performance

The MXene and nanoparticles loading on the modified electrode were optimized for optimal performance using impedance spectroscopy. Initially, five different MXene concentrations ranging from 1-5 mg/mL were selected for the fabrication of GCE/MXene and tested for impedance analysis. As illustrated in Fig. S6a, the results indicated an optimal MXene concentration of 4 mg/mL for decreased charge transfer resistance. Consequently, a concentration of 4 mg/mL was chosen for further modifications to the electrode. Subsequently, the concentration of PB NPs was optimized (Fig. S6b) on GCE/MXene/PB NPs, and a concentration of 2 mg/mL was found to provide a lower impedance for the modified electrode. Similarly, the loading of AuNPs was optimized, and AuNPs loading of 2 μL was selected for the final electrode modification (Fig. S6c). Regarding Nafion, it was observed that an increase in concentration led to a drastic increase in impedance. Hence, a lower concentration of 0.1% was chosen for the fabrication of the Kojic acid sensor.

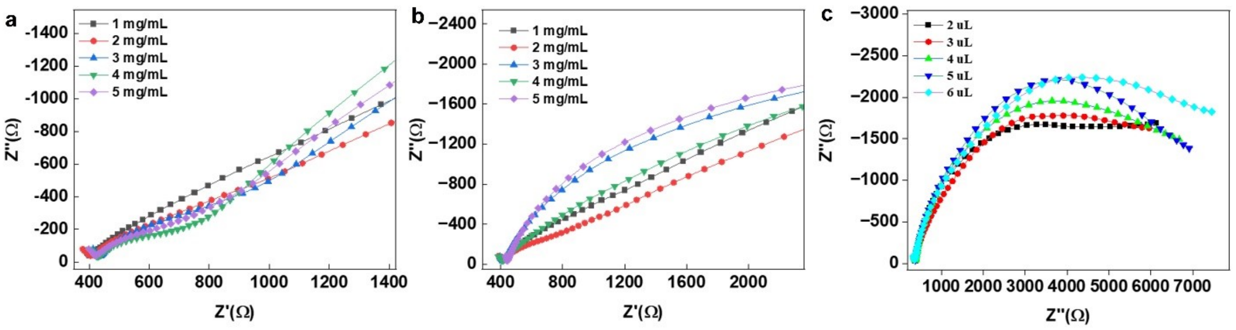

Fig. S6 Optimization of (a) MXene concentration, (b) PB NPs, and (c) AuNPs. The experiment was performed in 5 mM $K_4Fe(CN)_6$ and KCl supporting electrolyte.

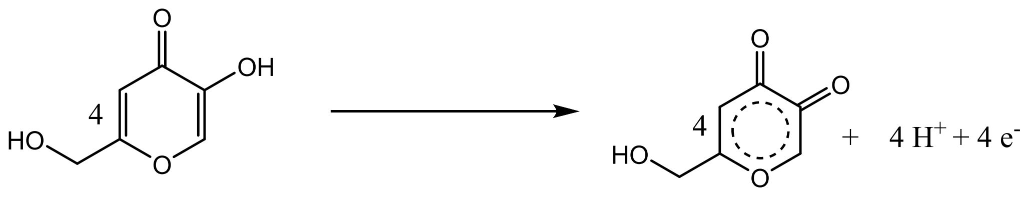

$Fe^{III}_4[Fe^{II}(CN)_6]_3 + 4K^+ + 4\ e^- \longrightarrow K_4Fe^{II}_4[Fe^{II}(CN)_6]_3$

Prussian blue, blue — Prussian white, colorless

Fig. S7 A plausible mechanism for the ratiometric electrochemical sensor for Kojic acid. Adapted from[3,4].

Real sample analysis

Real sample analysis was carried out using Kojic acid soap and apple cider vinegar. Briefly, the Kojic acid soap was ground into fine powder. 0.5 g of the ground soap was transferred into a beaker and dissolved in 100 mL PBS (pH 7.4) by gentle heating. The solution was centrifuged to remove any particulate matter. pH of the solution is adjusted using dilute HCl. Known concentrations of standard Kojic acid were then introduced into the solution by the standard addition method and stored at 4 ^{o}C. Apple cider vinegar was purchased and diluted 100 times using PBS 7.4. Known concentrations of standard Kojic acid were introduced into the solution by the standard addition method and stored at 4 ^{o}C.

References

1 M. Ivanovskaya, E. Ovodok, D. Kotsikau, I. Azarko, M. Micusik, M. Omastova and V. Golovanov, *RSC Adv.*, 2020, **10**, 25602–25608.

2 B. Ahmed, D. H. Anjum, M. N. Hedhili, Y. Gogotsi and H. N. Alshareef, *Nanoscale*, 2016, **8**, 7580–7587.

3 L.-C. Chen, T.-C. Liao, S.-F. Hong and C.-H. Chang, 2014.

4 S. Avazpour, A. Pardakhty, E. Nabatian and S. Ahmadzadeh, *Bionanoscience*, 2020, **10**, 502–511.
